# Matrix is everywhere: extracellular DNA is a link between biofilm and mineralization in *Bacillus cereus* planktonic lifestyle

**DOI:** 10.1038/s41522-023-00377-5

**Published:** 2023-02-28

**Authors:** Lyubov A. Ivanova, Vladimir V. Egorov, Yana A. Zabrodskaya, Aram A. Shaldzhyan, Alexander Ye. Baranchikov, Natalia V. Tsvigun, Anna N. Lykholay, Alexey D. Yapryntsev, Dmitry V. Lebedev, Anna A. Kulminskaya

**Affiliations:** 1 Petersburg Nuclear Physics Institute named by B.P. Konstantinov of National Research Center “Kurchatov Institute”, 1, mkr. Orlova roshcha, Gatchina, Leningradskaya oblast Russia; 2 Kurchatov Genome Center - PNPI, 1, mkr. Orlova roshcha, Gatchina, Leningradskaya oblast Russia; 3 Institute of Experimental Medicine, 12 Akademik Pavlov str., St. Petersburg, 197376 Russia; 4 Peter the Great Saint Petersburg Polytechnic University, 29 Polytechnicheskaya str., St. Petersburg, 194064 Russia; 5 Smorodintsev Research Institute of Influenza, Russian Ministry of Health, 15/17 Prof. Popova str., St. Petersburg, 197376 Russia; 6 Kurnakov Institute of General and Inorganic Chemistry of the Russian Academy of Sciences, 31, Leninsky av., Moscow, 119071 Russia; 7 Federal Scientific Research Center “Crystallography and Photonics” of the Russian Academy of Sciences, 59, Leninsky av., Moscow, 119333 Russia; 8 Centre for Molecular and Cell Technologies, Research Park of Saint-Petersburg State University, Petergof, 17, Botanicheskaya str., Saint-Petersburg, Russia

**Keywords:** Biological sciences, Microbiology, Biofilms

## Abstract

To date, the mechanisms of biomineralization induced by bacterial cells in the context of biofilm formation remain the subject of intensive studies. In this study, we analyzed the influence of the medium components on the induction of CaCO_3_ precipitation by the *Bacillus cereus* cells and composition of the extracellular matrix (ECM) formed in the submerged culture. While the accumulation of extracellular polysaccharides and amyloids appeared to be independent of the presence of calcium and urea during the growth, the accumulation of extracellular DNA (eDNA), as well as precipitation of calcium carbonate, required the presence of both ingredients in the medium. Removal of eDNA, which was sensitive to treatment by DNase, did not affect other matrix components but resulted in disruption of cell network formation and a sixfold decrease in the precipitate yield. An experiment with a cell-free system confirmed the acceleration of mineral formation after the addition of exogenous salmon sperm DNA. The observed pathway for the formation of CaCO_3_ minerals in *B. cereus* planktonic culture included a production of exopolysaccharides and negatively charged eDNA lattice promoting local Ca^2+^ supersaturation, which, together with an increase in the concentration of carbonate ions due to pH rise, resulted in the formation of an insoluble precipitate of calcium carbonate. Precipitation of amorphous CaCO_3_ on eDNA matrix was followed by crystal formation via the ACC-vaterite-calcite/aragonite pathway and further formation of larger mineral aggregates in complex with extracellular polymeric substances. Taken together, our data showed that DNA in extracellular matrix is an essential factor for triggering the biomineralization in *B. cereus* planktonic culture.

## Introduction

The process of microbially induced calcium carbonate precipitation (MICP) has received a lot of attention during the last decade. One of the most intensively studied applications of MICP is the development of technologies that use calcifying microorganisms to restore concrete structures. In most cases, the microbiological method has been developed for the restoration of historical stone buildings^1,2^, The main advantage is considered to be the imitation of the natural mineral formation that should guarantee good compatibility of the newly formed CaCO_3_ mineral and the building material^3,4^, The importance of studying biomineralization processes induced by microorganisms is also evident in areas related to human health. In clinical practice microorganisms with high urease activity, such as *Proteus mirabilis*, can form an encrusted biofilm that clogs urinary catheters often causing urinary retention and ascending infection of urinary tract, which can lead to cystitis, pyelonephritis, development of bladder stones, kidney stones or even fatal complications: sepsis and endotoxic shock^4^. Seifan et al. reported the use of MICP in the restoration of microcracks in tooth enamel^5^. In all cases, it is bacteria that play the main role in these processes and, therefore, understanding the mechanisms of the microbial induction of mineralization remains decisive for both civil engineering and biomedical applications.

One of the most popular hypotheses for the biogenic mineralization is crystal formation on microbial membranes. Several research groups reported that intracellular carbonate ion formation during cell metabolic reactions together with free calcium ions attraction outside the cell were followed by the formation of CaCO_3_ precipitates at the negatively charged cell wall surface, as reviewed in ref. ^6^. In this scenario, the crystal morphology depends on many factors: the bacterial cell morphology, environmental components, as well as physical factors (temperature, pH, and aeration)^7^. This was confirmed by Ghosh et al. who demonstrated the appearance of nanoscale calcium carbonate crystals at the surface of *Sporosarcina pasteurii*^5^ supporting the hypothesis of the central role of a negatively charged surface in the formation of crystals. In contrast, Zhang et al. claimed that a negatively charged surface was not a necessary factor for crystal precipitation and the bacterial role in this process is more complicated^6^. Bundeleva et al. have observed no crystal formation on the surface and near the cell in the studies on anoxygenic *Rhodovulum* sp.^7^. The authors hypothesized that there are mechanisms of cell protection against mineral deposition and proposed the idea of crystal deposition at some distance from the cell surface. Zhang et al.^8^ suggested that there is a spherical core formed at the initial stage followed by the formation of a crystal structure of various shapes depending on the growth conditions. The amorphous phase of CaCO_3_ (ACC) apparently could play an important role in this process, being one of the main components of the spherical core^9^. Thus, the principal role of bacterial cell wall as a calcium carbonate bioprecipitation point remains debatable and the exact mechanisms for the biomineralization process still require more detailed investigation.

In their recent review, A. Keren-Paz and I. Kolodkin-Gal detailed the accumulating evidence for the important role of the mineral inorganic component of the multicellular community and questioned the notion that bacterial-associated mineralization is a passive, unintended byproduct of bacterial metabolism^10^. The biomineralization mechanisms therefore could be viewed in a broader context of self-organization of bacterial community, such as biofilm formation. Recently, a team of these researchers has convincingly shown that calcium carbonate mineralization is a biologically regulated conservative process that affects the development of biofilms^11^. Biofilm is a common way for single-celled organisms to exist within a multicellular community by the formation of an extracellular matrix (ECM), which mainly includes proteins, extracellular DNA, and polysaccharides. ECM performs many functions ranging from cell protection and mechanical bonding of cells to providing an environment for “communication” of cells among themselves^12^.

Today it becomes clear that both processes, biofilm formation, and MICP, are complexed mutual processes, with numerous factors influenced on each and interplay between them. Hofmann et al. showed effect of several genetic and environmental factors on the calcification rate, yield and isoforms of the resulting minerals induced by *B. subtilis*^13^. In one of the latest works, it was shown that the morphology and structure of biogenic calcium carbonate minerals depend on such components of the extracellular matrix as proteins and polysaccharides^14^. Extracellular DNA appears to be an important integral component of ECM in *B. licheniformis*, being a stabilizing factor for proteins and amyloidogenic filaments thus affecting the biofilm cohesion^15^, and to provide cell adhesion in biofilm-forming *B. cereus*^16^. However, the extent to which eDNA and the process of biomineralization are related to each other remains unclear.

For our work, we chose widely distributed in nature the Gram-positive bacterium *B. cereus* as a model microorganism with high urease activity and tolerance to elevated calcium and urea concentrations available in the laboratory collection^17^. We have demonstrated the effect of the composition of the basic mineralizing medium on the behavior of bacterial cells and their ability to induce mineralization. We have shown the role of eDNA as a potential driver of biomineralization. These data may be useful for the future development of engineered living materials and other applications that is possible with careful regulation of the MICP process.

## Results

**Fig. 1:**
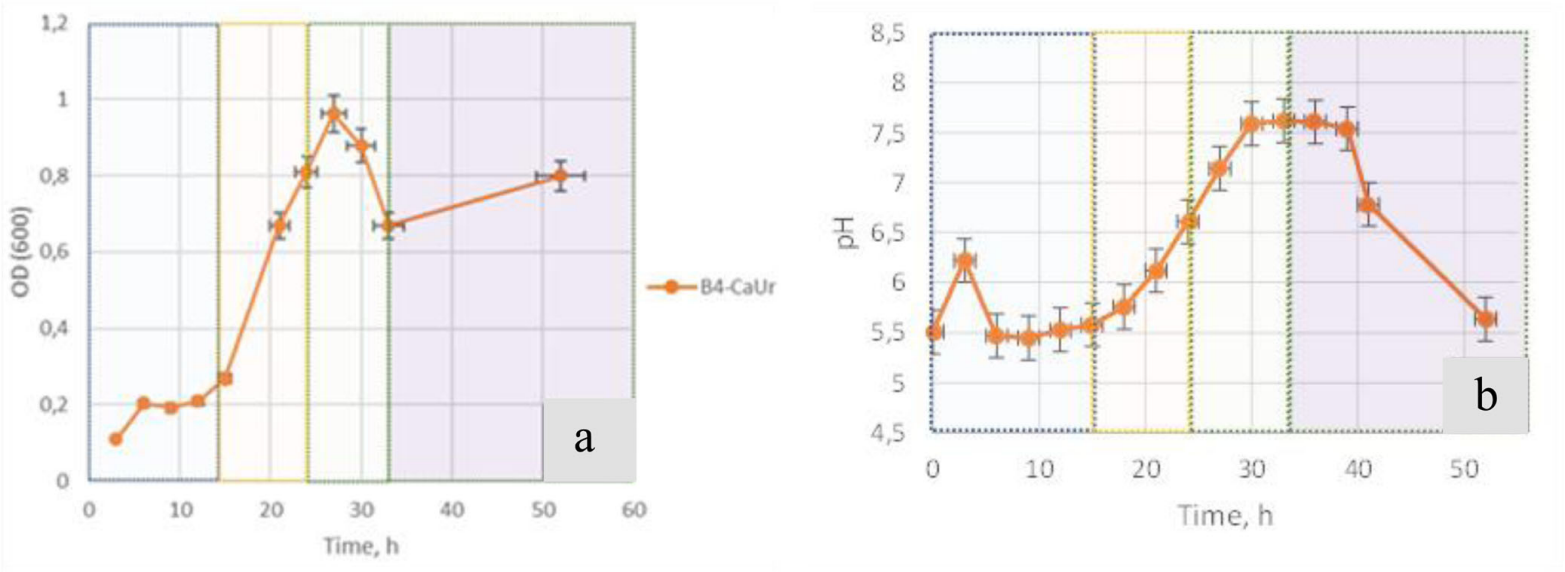
Biomass growth characteristics of *B. cereus* 4B in the B4_CaUr medium (with an excess of calcium and urea ions). **a** Time-dependence of the bacterial biomass growth; **b** Time-dependence of the medium acidity during the incubation. Time stages are highlighted in color: 0–15 h in blue; 15–24 h in orange; 24–33 h in green; more than 33 h in lilac.

*B. cereus* growth curves measured in four different media are shown in Fig. 1a and S1. In the media containing Ca^2+^ the exponential growth was preceded by a lag phase, and the overall rate of biomass accumulation was somewhat lower than in the Ca^2+^-free media with a plateau reached by 30–35 h of cultivation. Under Ca^2+^-free conditions in both media the bacteria showed similar exponential growth curves without lag phase reaching a plateau after 10–15 h of cultivation (Supplementary Fig. 1). By the end of the second cultivation day, the cell density had approximately the same value in each of the four media used. According to microscopic data intensive mineral formation was observed in **B4_CaUr** medium after 24-h growth. No mineral formation was seen in either urea-free (**B4_Ca**) or in the Ca-free (**B4_Ur**) media.

In accordance with the dynamics of bacterium biomass accumulation (Fig. 1a), changes in pH (Fig. 1b) and the key mineralization events the process of mineral formation by *B. cereus* 4B in planktonic culture observed in Ca^2+^- and urea-supplemented medium could be divided into 4 stages

### Stage 1 (0–15 h)

**Fig. 2:**
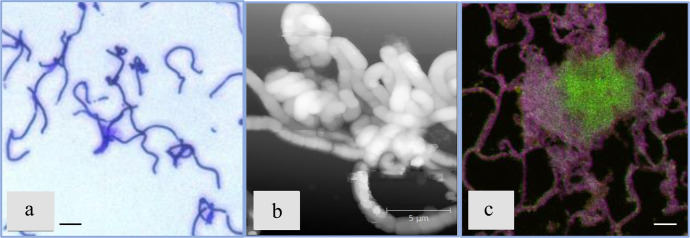
*B. cereus* 4B at the first stage of the growth (0–15 hours after inoculation). **a** Light-microscopy image with crystal violet staining in the sample taken after 9 hours after the inoculation. **b** AFM microscopy image (after 15 h). **c** Confocal microscopy image with Sytox green staining (green color, specific for eDNA), Congo Red (pink color, amyloids and polysaccharides) and CaCO_3_ autofluorescence channel (yellow color); All scale bars correspond to 5 µm.

The growth curve of *B. cereus* cells in the basic (**B4_CaUr**) medium was characterized by a lag phase (Fig. 1a) that included an initial increase in pH and subsequent decrease to the initial pH value after an hour of cultivation (Fig. 1b). An elongation of the lag phase and a similar increase in pH was also observed in the **B4_Ca** environment (Supplementary Fig. 1). Extracellular structures within the clusters of bacterial cells visualized by diffuse staining with crystal violet, a classic stain for biofilm detection^18,19^, were seen as early as 6 hours into the bacterial growth (Fig. 2a). Similar structures appeared in the medium **B4_Ca** (with the excessed calcium without urea) and were absent in the other controls (in the media **B4_Ur** and **B4**, Supplementary Fig. 2). By 15 hours of growth in the presence of urea and Ca^2+^ (**B4_CaUr** medium), the cells began to form so-called “curls”^20^, which were clearly seen by AFM (Fig. 2b). By the end of the first stage of the growth we also detected diffuse autofluorescence of calcium carbonate precipitate^21^ that was co-localized with the extracellular DNA staining by specific fluorescent dye Sytox green (Fig. 2c), which was only seen in **B4_CaUr** medium.

### Stage 2 (15–24 h)

During the second stage of cultivation in the **B4_CaUr** medium we observe exponential growth of bacterial biomass (Fig. 1a) and sharp alkalization of the medium (Fig. 1b). A similar change in pH of the culture medium was observed in the Ca-free **B4_Ur** medium and was absent in other controls (Supplementary Fig. 3). Medium alkalization indicates the active participation of urea in the metabolism of *B. cereus* 4B demonstrating the presence of urease activity that resulted in ammonium formation.

**Fig. 3:**
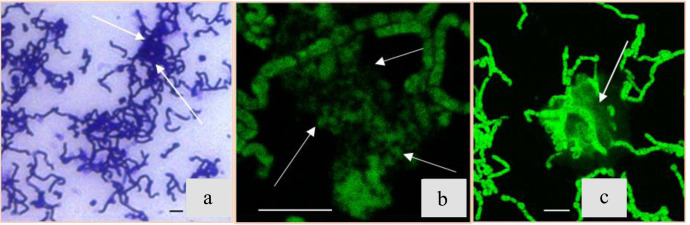
*B. cereus* at the second stage of the growth (15–24 h after inoculation). **a** Light-microscopy image with crystal violet staining in the sample taken after 24 h after inoculation; white arrows show ECM thickening. **b** Confocal microscopy image with Sytox green staining (eDNA). **c** Confocal microscopy image with Thioflavin T staining (amyloid structures); All scale bars correspond to 5 µm.

By the 24th hour, we observed the formation of cell aggregates among most bacterial cells in culture, which was visible under light microscopy of samples stained with crystal violet (Fig. 3a, shown by arrows). The areas of cell consolidation subsequently comprise the locations where most of CaCO_3_ precipitates were deposited. During this stage the amount of eDNA (observed in **B4_CaUr** medium by staining with Sytox green, Fig. 3b) has increased and amyloid structures diffusely stained with the fluorescent dye Thioflavin T^22^ have appeared (Fig. 3c).

### Stage 3 (24–33 h)

The third stage of the bacterial growth in the **B4_CaUr** medium was characterized by a significant decrease in the optical density (Fig. 1a), which did not take place in other controls (Supplementary Fig. 1). By 30 h after inoculation, the medium pH reached a plateau (Fig. 1b). By comparison, in Ca^2+^-free **B4_Ur** medium the pH kept increasing. Lower alkalization in the presence of calcium was likely caused by absorbing some of the carbonate ions by Ca^2+^ during biomineralization reactions.

**Fig. 4:**
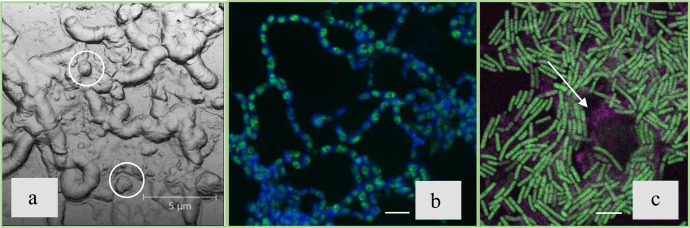
*B. cereus* 4B at the third stage of the growth (24–33 h after inoculation). **a** AFM image of the sample taken at 33 h after inoculation. **b** Confocal microscopy image with Sytox green (eDNA, green color) and Bromophenol blue (amyloid structures, blue color) staining. **c** Confocal microscopy image staining with Thioflavin T (amyloid structures, green color) and Congo red (amyloid structures and/or polysaccharides, pink color); All scale bars correspond to 5 µm.

Microscopic observations have revealed the appearance of submicron crystalline precipitates of calcium carbonate localized in the extracellular matrix. Dense calcium carbonate aggregates reached a size of several microns by the end of the third stage (33 h after inoculation) (Fig. 4a, marked with white circles).

Staining the ECM of the **B4_CaUr** medium samples with fluorescent dyes Sytox green (eDNA) and Bromophenol blue (specific dye for proteins^23^) revealed differentiation of cells within the community (Fig. 4b). A similar observation was also characteristic for other pairs of dyes: Thioflavin T and Congo red (Fig. 4c). The latter dye can bind not only amyloid structures but also some polysaccharides^24^, which accounted for diffuse staining near precipitates (Fig. 4c, shown by arrows).

### Stage 4 (more than 33 h)

**Fig. 5:**
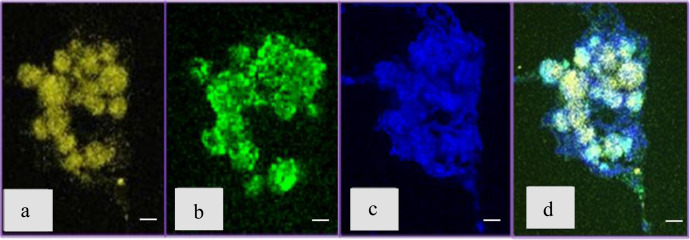
*B. cereus* 4B at the fourth stage of the growth (45 h after inoculation). **a** Confocal microscopy image with CaCO_3_ autofluorescence. **b** Confocal microscopy image with Sytox green (eDNA) staining. **c** Confocal microscopy image with Bromophenol blue staining (amyloid structures). **d** Confocal microscopy image with Sytox green (eDNA, green color), Bromophenol blue (amyloid structures, blue color) and CaCO_3_ autofluorescence (yellow color); All scale bars correspond to 5 µm.

The fourth stage of cultivation in the **B4_CaUr** medium was characterized by a stabilization in bacterial biomass (Fig. 1a) and a decrease in pH (Fig. 1b) until the end of the observations. After 33 h of inoculation, the size of some CaCO_3_ aggregates reached several tens of microns and started to form large agglomerates (Fig. 5a) in the extracellular matrix. The eDNA was primarily localized inside the precipitates (Fig. 5b) which were partially incorporated in the surrounding protein and amyloid structures (Fig. 5c, d).

**Fig. 6:**
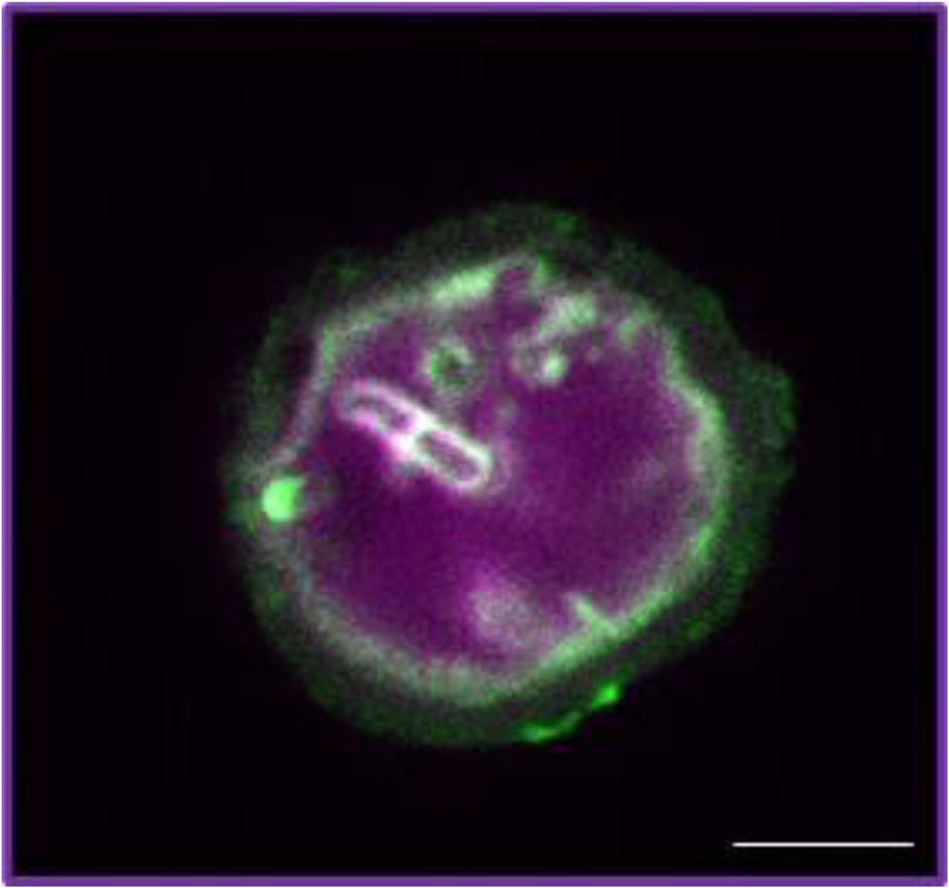
Confocal microscopy image of *B. cereus* 4B at the fourth stage of the growth (52 h after inoculation). DNA was stained with 7AAD (pink color) and amyloid structures were stained with Thioflavin T (green color). All scale bars correspond to 5 µm.

Staining of the large mineral aggregates with 7AAD (DNA-specific dye) together with Thioflavin T (amyloid-specific dye) indicated a presence of eDNA inside the aggregate together with some cell inclusions, and distinct amyloid shell on the mineral surface (Fig. 6).

The conditions of bacterial growth had a significant effect on the time scale of cell sporulation. In the **B4_CaUr** medium, where biomineralization was observed, non-sporulating cells were seen on microscopic images as late as 78 hours of cultivation, mainly in ECM clusters with CaCO_3_ agglomerates (analogically Fig. 5). In urea-rich Ca-free medium the sporulation was observed after 50 h of cultivation (Supplementary Fig. 4a), while the sporulation process in the urea-free medium had taken place even earlier, at around 40 h of cultivation (Supplementary Fig. 4b.). We also used dead, treated with gamma radiation, bacteria as controls to ensure that CaCO_3_ precipitation are indeed caused by live cells. The experiment with dead *B. cereus* cells resulted in no mineralization (data not shown).

### Precipitate mineral analysis

**Fig. 7:**
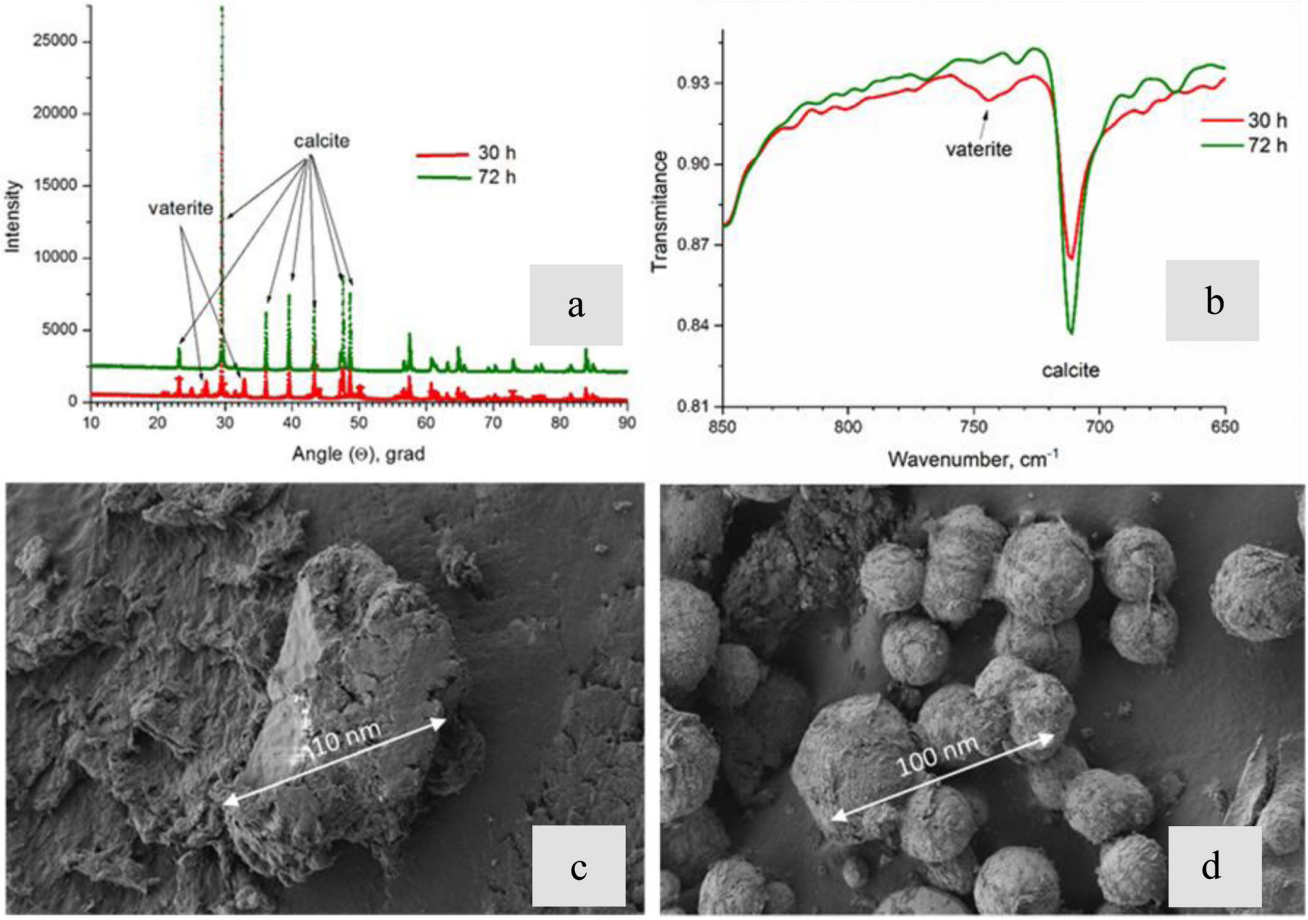
Mineral analysis of CaCO3 precipitates induced by the *B. cereus* during the growth in the B4-CaUr medium. **a** X-ray diffraction patterns of the samples shows vaterite peak loss after 30 h. **b** FTIR spectra of precipitates at different times of growth shows polymorph transition from vaterite to calcite. **c**, **d** SEM images of CaCO_3_ particles induced by *B. cereus* at 24 and 72 h after inoculation, correspondingly.

Samples with CaCO_3_ precipitates obtained during the growth of the bacterium in the Experimental media were analyzed by XRD and FTIR methods (Fig. 7a, b). Intense absorption bands of the carbonate ion (ν_1–4_ CO_3_^2−^), OH-groups and water^25^ can be easily distinguished on the FTIR spectra of the obtained samples (Fig. 7b). Low-intensity bands with maxima at 1800 and 2500 cm^–1^ can be attributed to the composite vibrational frequencies of the carbonate ion or organic impurities (ketones, thiols, or amines)^26^. Intense bands ν_2–4_ CO_3_^2−^ (~710, ~870, and ~1400 cm^−1^) corresponded to the most abundant calcite phase in the obtained samples. However, the presence of the ν_1_ CO_3_^2−^ band (~1080 cm^−1^) indicated the presence of small impurities of aragonite, vaterite, or amorphous calcium carbonate (ACC) in the studied samples^26,27^, The band with a maximum of ~750 cm^–1^ has been found only in the spectra of samples collected at point 30 h after the inoculation indicating the presence of vaterite^27,28^, in their composition. The amorphous phase is usually characterized by the ratio of the ν_2_ to ν_4_ band intensities for the carbonate ion^29^. Accordingly, this ratio increased in the samples taken at 30 h after inoculation and reached a maximum for samples containing traces of vaterite phase. Thus, the data of FTIR spectroscopy were in a good agreement with the known model of calcium carbonate polymorphs transformation: ACC → vaterite → calcite, aragonite^29^.

The X-ray diffraction analysis (Fig. 7a) revealed changes in the distribution of calcium carbonate polymorphs during the microorganism growth. Therefore, for precipitated samples washed out from the culture liquid at the point 30 h after the *B. cereus* inoculation, a noticeable amount of vaterite, consisting about 10% of the total content of calcium carbonate crystals, was detected. Further, the vaterite phase gradually transformed into calcite so that in the samples at 72 h after the inoculation the vaterite peaks were no longer detected. Accumulation of calcite phase was accompanied with ACC disappearance detected by scanning electron microscopy (Fig. 7c). At the point of 30 h after the inoculation small spherulites (50–70 μm in size) with bacterial cell traces on their surfaces were located among the amorphous phase as seen in the sample microphotographs (Fig. 7c). After 72 h of cultivation, the inorganic precipitates increased in size reaching a structure with an average diameter from 300 to 500 μm (Fig. 7d). Noteworthy, the latter precipitates had better crystal structure than their 30-h precursors. During the following period, the precipitates hardly increased in size, their structures looked crystalline, their amorphous environment disappeared completely (Fig. 7d).

### Effect of DNase (I) on the ECM content and mineralization

To study a connection between eDNA component of ECM and biomineralization, we used DNase (I) to remove the eDNA from ECM^30,31^, In the preliminary experiment, the activity of the DNase (I) in the medium with lower concentrations of urea and Ca^2+^ ions in the **B4** medium (2.5 g/L of urea and 2.5 g/l CaCl_2_ vs 25 g/L of urea and 25 g/l CaCl_2_ in the basic **B4_CaUr**) was assayed. The experiment revealed the enzyme was active at digesting salmon sperm DNA into small fragments which was confirmed by PAGE (Supplementary Fig. 5a). It was also shown in situ by confocal microscopy with Sytox green that the addition of DNase almost eliminated the DNA component from the ECM after one hour of incubation (Supplementary Fig. 5b–e).

**Fig. 8:**
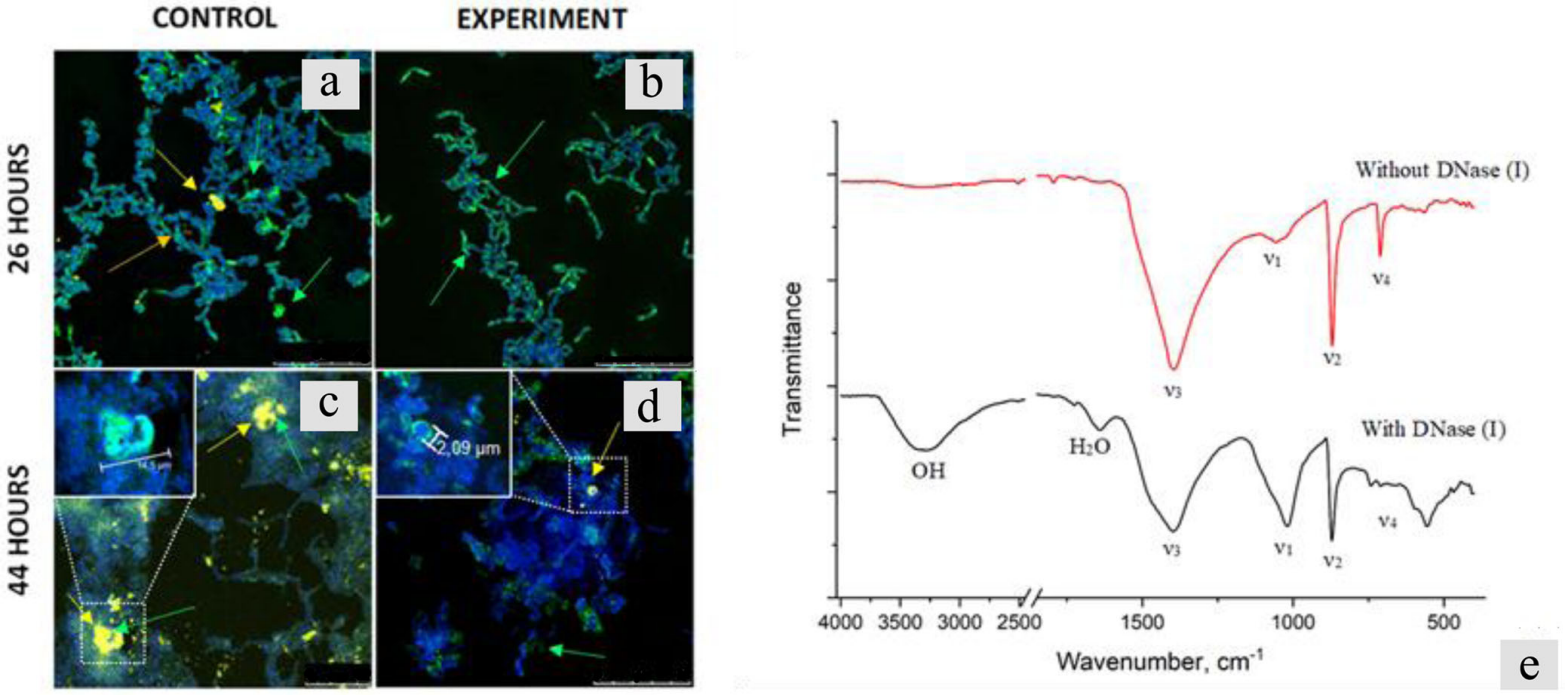
Results of the eDNA removal from the extracellular matrix of the *B. cereus* planktonic culture. Confocal microscopy images with Sytox green (eDNA, green color), Bromophenol blue (protein structures, blue color), and CaCO_3_ autofluorescence (yellow color) staining of *B. cereus* samples cultured in **B4_CaUr** medium without addition of DNase (I) (**CONTROL a**, **c**) and repeated addition of DNase (I) (**EXPERIMENT b**, **d**) for 26 h (**a**, **b**) and 44 h (**c**, **d**). **e** FTIR spectra of precipitates obtained during biomineralization of *B. cereus* in a medium with the addition of DNase (red line) and without DNase (black line). All scale bars correspond to 25 µm.

Figure 8 shows confocal images of the samples taken at 26 and 44 h of cultivation and stained with Sytox green (green color, eDNA) and Bromophenol blue (blue color, protein structures) fluorescent dyes. By the 26th hour of growth in the absence of DNase we saw diffusely stained eDNA (Fig. 8a, green arrow), as well as the formation of both diffuse (Fig. 8a, orange arrow) and condensed CaCO_3_ precipitates, detected by autofluorescence (Fig. 8a, yellow arrow). By the same time of growth in the DNase-supplemented medium we observed Sytox green to bind only to structures on the surface of bacterial cells (Fig. 8b, green arrow) and no diffuse staining or autofluorescence of calcium carbonate was detected (Fig. 8b). No significant effect of DNase on the localization and amount of protein structures (stained by Bromophenol blue) was observed at any stage of the experiment (Fig. 8a–d).

At 44 h of cultivation in the **B4_CaUr** medium without DNase, agglomerates of calcium carbonate reached the sizes of tens of microns (Fig. 8c, yellow arrow). In the media where the DNase was added we detected only individual small accumulations of eDNA (Fig. 8d, green arrow), as well as CaCO_3_ precipitates, which reached the size of several microns. The appearance of eDNA likely indicated that the enzyme was inactivated for 11 h following the addition of the last portion of the DNase to the experimental sample (at 33 h), either by urea or by other cell metabolites present in the medium. In contrast with the results shown in the previous section, eDNA, which in the absence of DNase was seen inside the calcium carbonate agglomerates (Fig. 8c, green arrow), was localized at the periphery of the mineral formations, while the interior of the precipitate aggregates contained proteins stained by Bromophenol blue (Fig. 8d, yellow arrow, enlarged left and Supplementary Fig. 8).

The weight of the visually differed precipitates in the medium with the addition of DNase was about 6 times less than those without DNase treatment (16 and 94 mg, respectively). In the IR spectra (Fig. 8e) of the obtained samples, one can distinguish intense absorption bands of the carbonate ion (ν_1–4_ CO_3_^2−^), as well as bands of hydroxyl groups and water^25^. Low-intensity bands with maxima at 1800 and 2500 cm^–1^ can be related to the composite vibration frequencies of the carbonate ion or impurity organic compounds (ketones, thiols, or amines)^26^. Intense bands ν_2–4_ CO_3_^2−^ (~710, ~870 and ~1400 cm^−1^) correspond to the calcite phase. The ν_1_ CO_3_^2−^ band (~1080 cm^–1^) indicates the presence of aragonite, vaterite, or amorphous calcium carbonate impurities in the obtained samples^27,28^, The presence of vaterite can be additionally confirmed by the presence of an absorption band with a maximum of ~750 cm^−1,^^28,29^^,^ Such a band is present only in the spectrum of the sample with the addition of DNase (Fig. 8e, black line), which indicates the presence of vaterite in their composition. The ν_1_ CO_3_^2−^ band (~1080 cm^−1^) has the highest relative intensity also for the sample with the addition of DNase, which indicates the highest content of impurities in this sample. In addition, the IR spectroscopy data for this sample indicate the presence of a large amount of water in its composition. In the region of 3000–3700 cm^−1^, a wide band of stretching vibrations of OH is observed, at ~1600 cm^−1^, a band of bending vibrations of HOH, and in the region of 500–600 cm^−1^, bands of librational vibrations of crystallization water^25^.

The content of the amorphous phase in the literature is usually estimated from the ratio of the intensities of the ν_2_ to ν_4_ bands for the carbonate ion^8^. Accordingly, the proportion of ACC is also higher in the sediment sample obtained by adding DNase to the medium.

**Fig. 9:**
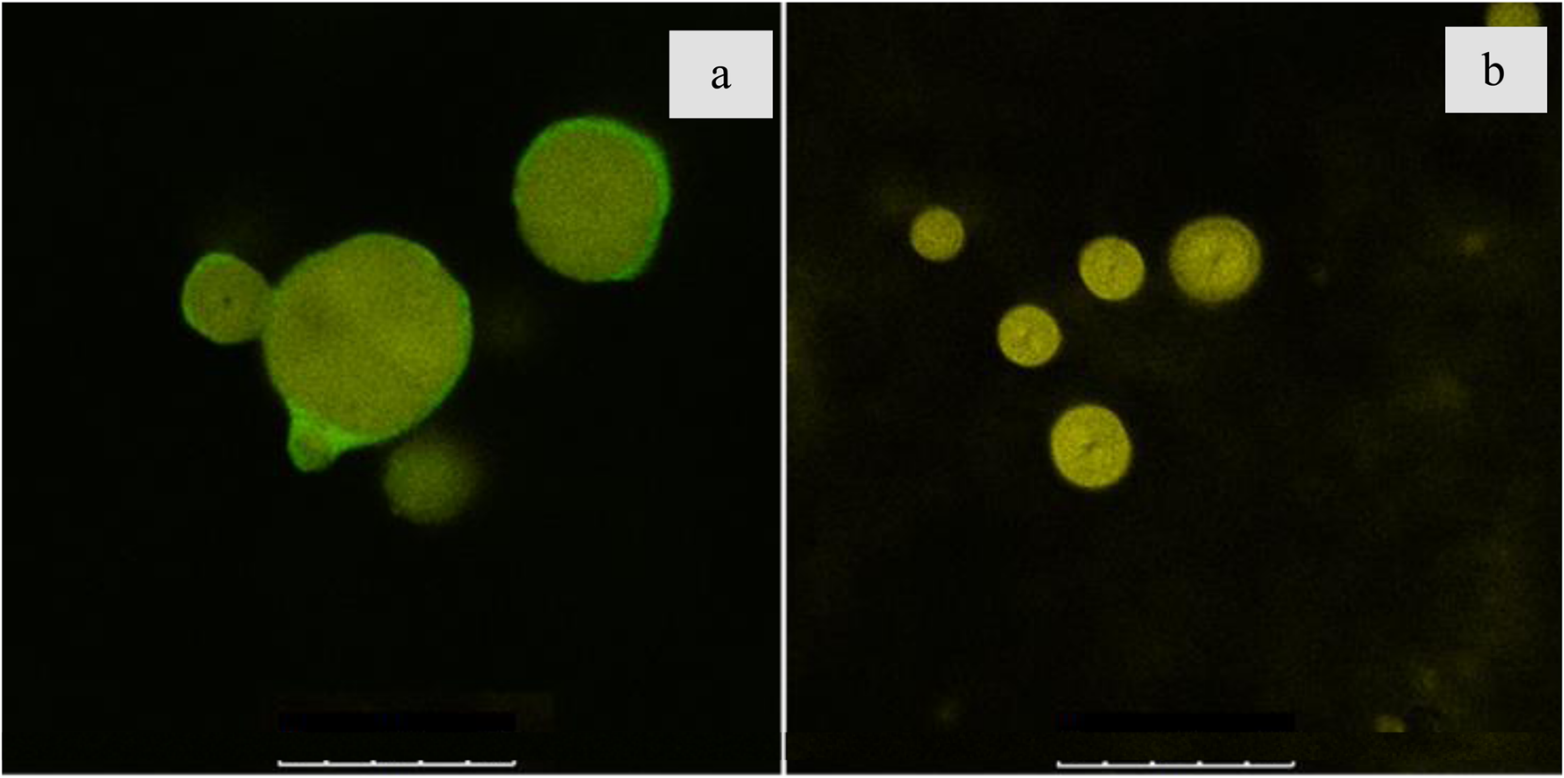
Confocal microscopy images of CaCO3 precipitates deposited in vitro. **a** With exogenous DNA stained with Sytox green; **b** Without eDNA stained with Sytox green. All scale bars correspond to 25 µm.

We used cell-free system to analyze the role of DNA in the process of biomineralization. For this, exogenous DNA from salmon sperm and CaCl_2_ were added to the culture supernatant of *B. cereus* in **B4_Ur** medium obtained at the stage when cultivation pH reached 8. This point was chosen as previously observed start for CaCO_3_ precipitation induction by *B. cereus* cells. Precipitation in the control sample without exogenous DNA was observed only after 30 minutes, while in the experimental sample with salmon sperm DNA, CaCO_3_ precipitation was visually noticeable already after 5 min at 37 °C. Figure 9 shows confocal images of CaCO_3_ precipitates produced by cell-free system with and without exogenous DNA. Sytox green-stained DNA fluoresced inside the calcium carbonate precipitate (Fig. 9a) indicating that it was formed on DNA matrix while without exogenous DNA Sytox green fluorescence was not observed (Fig. 9b). Two hours after the start of the experiment the monitored spectrophotometrically^32^ amounts of precipitated calcium carbonate reached similar values.

## Discussion

By applying different composition of the cultivation media and monitoring the processes throughout the bacterial culture growth we evaluated ECM content and evolution, as well as the possible role of its three major components (polysaccarides, eDNA and protein) may play in the mineral induction by *Bacillus cereus* in planktonic culture. In agreement with the earlier works (reviewed in ref. ^33^), the high calcium concentration appeared to be a stress factor for the bacterial cells. The cells responded to this stress factor by secretion of exopolysaccarides, which could chelate the excess of the Ca^2+^ ions. This was supported by the observation of the overall lower growth rate and increased lag phase in the Ca^2+^-rich medium, as well as by formation of clouds around the cells visible by AFM (Supplementary Fig. 6a, black arrow) and diffuse staining of the surrounding areas by crystalline violet (Supplementary Fig. 6b, black arrow) but not by DNA- or protein-specific dyes.

Urea, while commonly known for its bacteriostatic action^34^, can be utilized by some bacterial species as a source of nutrition via a nitrogen metabolism pathway by promotion of the synthesis of ureоlytic enzymes^35^. The presence of urea in the growth medium of *B. cereus* 4B resulted in an increase in the overall rate of biomass. We also have noted that despite a faster growth rate, spore formation by the bacteria in urea-rich medium was observed after 52 h of incubation, as compared to 33 h in the Ca^2+^- and urea-free medium.

Precipitation of calcium carbonate was observed in the calcium and urea-rich (**B4_CaUr**) medium but not in the media with only calcium (**B4_Ca**) or only with urea (**B4_Ur**). This indicates that Ca^2+^ and carbonate ions, formed as a result of intracellular metabolic reactions, are not sufficient either for the nucleation of CaCO_3_ crystals inside the cell and their subsequent secretion, as previously assumed^6–9^, nor for their formation in the extracellular space, thus implying involvement of other factors in the mechanisms of biomineralization. Indeed, AFM images clearly illustrate the fact that the growth of biogenic crystals occurred directly in the extracellular matrix, in large clusters of cells. Using a fluorescent dye Congo red, we observed the morphology of the cells in such agglomerates (Figs. 3c and 4c). Congo Red did not stain the bacterial wall but was detected in structures that covered the cells, observed in AFM topograms, which were probably comprised of functional amyloids reported contributing to formation of *curli pili* in *B. subtilis*, a close relative of *B. cereus*^27^, and implicated in such physiological tasks as biofilm formation, adhesion, host cell invasion, and host-pathogen interactions.^43^ Amyloid structures stained with Congo Red were largely incorporated in the extracellular matrix. Comparison of staining with amyloid-specific dye Thioflavin T and crystalline violet indicated that exopolysaccarides, which appeared in the Ca^2+^-rich medium, could act as the sites of initiation for the formation of amyloid-like structures by proteins of the extracellular space (Supplementary Fig. 6).

In our experiments we have detected an extracellular metalloprotease M4 (see the details in the Supplementary methods, notes and references, Supplementary Fig. 9)^36^, which could participate in the modification of matrix proteins possibly inducing conformational changes and formation of amyloids. Amyloid formation was previously shown for glyceraldehyde-3-phosphate dehydrogenase^37^. We were able to detect this enzyme (Supplementary Fig. 9), as well as to observe the formation of amyloid-like extracellular structures, in all samples regardless of external conditions. It can be supposed that in the case of *B. cereus* 4B this particular protein, in addition to its enzymatic function, may play the role in building ECM by amyloid-like structure formation.

Besides the role in formation of the protein component of ECM, glyceraldehyde-3-phosphate dehydrogenase has been shown to act as a regulator of biofilm formation in a representative of *B. cereus* by triggering the formation of extracellular DNA^38^. In the calcium- and urea-free medium **B4**, we observed eDNA in relatively small quantities and only at the later stages of the growth. However, when both urea and calcium were added to the medium, substantial amounts of the eDNA component in the matrix appeared in the early stages of exponential growth and were increasing in time. In the **B4_Ur** medium we saw a moderate amounts of eDNA after 30 h of cultivation when the exponential growth phase was finished, while in **B4_Ca** medium eDNA was observed in small amounts after 15 h of growth and did not show tendency for further accumulation (Supplementary Fig. 7). The fact that appearance of eDNA in the **B4_CaUr** medium coincided to entering the exponential growth phase clearly indicated that eDNA was secreted by actively dividing cells and not by cell lysis. This observation confirms the results obtained earlier for *B. cereus* ATCC 14579 that the origin of eDNA during the planktonic growth was not cell lysis^39^. At approximately the same time as eDNA secretion was detected, we observed the appearance of amorphous calcium carbonate by IR and XRD (Fig. 7), and diffuse calcium carbonate autofluorescence by microscopy (Fig. 2). No calcium carbonate precipitation was detected after cells were sterilized by gamma radiation at 10 h of growth (see details in the Supplementary notes2).

One of the distinguishing features of the biomineralization process, shown by confocal microscopy, was co-localization of calcium carbonate with eDNA. At the earlier stages, the colocalization of diffuse calcium carbonate autofluorescence and fluorescence of Sytox green staining eDNA indicates the genesis of a large amount of amorphous calcium carbonate among a large accumulation of extracellular DNA secreted by cells for formation of the ECM. At the later stages, the large CaCO_3_ mineral aggregates were seen to enclose eDNA and were covered with amyloids outside (Figs. 5 and 6).

In further evidence for the role of eDNA in this process, no mineralization occurred as long as the DNA component was removed from the matrix by DNase treatment. Unlike the previous reports suggesting that eDNA is tightly coupled to the protein matrix^40^, we saw little effect of applying DNase on the protein component of ECM in planktonic cells. However, the ability of cells to form aggregates in the absence of eDNA was reduced. The strong inhibition of mineral formation by DNase could be attributed to indirect effects of eDNA removal from ECM on the diffusion parameters of microenvironment in the cell clusters. Weaker ECM and smaller size of cell aggregates would reduce the local concentrations of ions so that oversaturation cease to occur. On the other hand, literature reports (e.g. refs. ^14^ and ^38^) together with our observation that exogenous DNA is capable of inducing precipitation in cell-free medium would suggest the direct involvement of the negatively charged eDNA molecules in Ca^2+^ recruitment and precipitation. At the same time, the contribution of microenvironment in ECM as well as involvement of amyloids in nucleation cannot be ruled out. After the addition of fresh DNase has ceased and the enzyme became degraded, a small amount of eDNA and few mineral particles up to micron-sized were formed in the samples (Fig. 8d). These particles contained proteins inside while еDNA was detected on their periphery that could indicate involvement of the protein component of ECM during some stages of precipitate evolution.

We therefore can propose the following mechanism for the formation of calcium carbonate crystals in *B. cereus* planktonic culture in a medium with an excess of calcium ions and urea: in the first stage (0–15 h of cultivation), cells protect themselves from stress conditions induced by elevated calcium concentrations by producing exopolysaccharides, which not only chelate calcium ions but also serve as sites for amyloidogenesis initiation of the extracellular proteins and thus contribute to the formation of ECM. Under these conditions cells agglomerate into “nests” where the formation of minerals begins, coinciding in time with the rise in the pH induced by cell metabolism in urea-rich environment. Extracellular DNA at this stage also appeared to be an important factor for triggering the biomineralization. In addition to its role in changing cell microenvironment by Ca^2+^ recruitment, ECM formation and cell clustering due to its negatively charged lattice-like structure^39^, eDNA could serve as a scaffold for suspended particulate organic and inorganic matter and thus promote formation of insoluble precipitate of calcium carbonate. By the end of the exponential growth stage (33 h) the cell clusters contained a large number of mineral particles reaching submicron sizes. The initially formed amorphous CaCO_3_ underwent the crystallization pathway from the ACC isomorph to vaterite and, further, to calcite (Fig. 7), while eDNA gets enclosed inside the large crystal aggregates, as seen by confocal microscopy using different DNA dyes (Figs. 5 and 6).

Biomineralization process in *B. cereus* thus appears to involve an interplay of multiple factors promoted by changes in bacterial cell metabolism and protein expression pattern in response to stress that lead to accumulation of extracellular protein amyloids and eDNA, which both participate in ECM formation. Under the conditions of elevated carbonate ion concentration caused by a combination of carbonic acid release and alkalization of the medium as a result of the cell metabolism in the presence of urea, these ECM components, particularly negatively charged eDNA, serve as inductors for precipitation of amorphous CaCO_3_ followed by crystal formation and mineral growth.

## Methods

### Bacterium growth conditions and sample preparations

The strain *Bacillus cereus* 4B (an undomesticated prototrophic strain from our laboratory collection)^19^ deposited at the National Bioresource Center “All-Russian Collection of Industrial Microorganisms” under the number B-14265 was maintained on LB medium (peptone 10 g/L, yeast extract 5 g/L and NaCl 10 g/L) containing 1% agar. To assess the influence of the medium components on the behavior of the *B. cereus* 4B planktonic cells during CaCO_3_ mineralization, four variants of standard B4 medium (yeast extract, 2 g/L; glucose, 10 g/L) were used: 1. control **B4** medium without urea and CaCl_2_; 2. B4 with the addition of urea (25 g/L) and without CaCl_2_ (**B4_Ur**); 3. B4 with the addition of CaCl_2_ (25 g/L) and without urea (**B4_Ca**); 4. B4 with urea and CaCl_2_ (**B4_CaUr**). Pre-sterilized solutions of urea or/and CaCl_2_ were added separately to the corresponding medium after autoclavation. The inoculum was preliminary grown in LB medium overnight and transferred into the 500 mL flasks containing 100 mL of the appropriate B4 medium and cultivated for 52 h at 37 °C with shaking at 110 rpm. Two-milliliters aliquots were withdrawn every 3 h followed by cooling to the room temperature (25 °C) for subsequent experiments. All growth experiments were repeated 5 times.

Biomass and pH changes during the cultivation were routinely monitored in all media by measurements of the culture liquid optical absorbance at 600 nm using spectrophotometer Hitachi U-3310 and pH using pH-meter (HANNA instruments HI8519N, USA) and pH-indicator strips (Merck KGaA, Germany).

### Microscopy

#### Light microscopy

Samples of ECM formed at different stages of cell growth were visualized using a Leica DM2500 optical fluorescence microscope after staining with crystal violet dye according to the method described in ref. ^18^ with minor modifications. Briefly: each sample was stained with 30 µL of a 0.1% water solution of crystal violet at room temperature for 3–5 min.

#### Atomic force microscopy

Bacterial surface was studied using an NT-MDT Solver Bio scanning probe microscope. Cells from each aliquot were applied to a glass slide and rinsed with MilliQ water for salt removal. Measurements were performed in a semi-contact mode using an NSG01 probe in parallel with visualization on SMEs-1 optical stereoscopic microscope.

#### Fluorescent microscopy

Bacterial cells, ECM and mineral precipitates formed at different stages of cell growth were visualized using confocal microscope Leica TCS SP5. Samples were placed on a microscopy glass, dried and sealed under the cover slip with glycerol. For the detection of extracellular DNA (eDNA) samples were stained with Sytox™ Green Ready Flow™ Reagent (Invitrogen, Thermo Fisher Scientific; fluorescence ex. 488 nm, em. 505–555 nm) and 7AAD (Invitrogen, Thermo Fisher Scientific; ex. 543 nm, em. 630–670 nm) according to the manufacturer’s instructions. To detect protein and amyloid structures, cells were stained with Thioflavin T (Sigma-Aldrich CO, USA; ex. 458 nm, em. 470–500 nm), Bromophenol blue (Sigma-Aldrich CO, USA; ex. 633 nm, em. 660–800 nm) and Congo Red ((Sigma-Aldrich CO, USA; ex. 514 nm, em. 600–650 nm). All dyes were diluted in water with a stock concentration of 1 mg/mL and used to stain the sample for 3–5 minutes. We also applied several stains for the same structures (such as Sytox green and 7AAD for eDNA) in order to exclude fluorescence from nonspecific binding and to be sure that the stained structures are eDNA or amyloid, respectively. Calcium carbonate was detected by autofluorescence (ex. 405 nm, em. 420–480 nm) according to the protocol^41^.

### CaCO_3_ particle analysis

To analyze CaCO_3_ particles, the precipitates were separated by centrifugation (4000 rpm, 5 min), washed with MilliQ water, dried at 37 °C in a thermostat and analyzed by powder X-ray diffraction, scanning electron microscopes and IR Fourier spectrometer. Powder X-ray diffraction (XRD) analysis of the samples was performed on a Rigaku440Miniflex 600 diffractometer (Bragg-Brentano geometry) with Ni-filtered CuKa (*λ* = 1.5418 Å) radiation and a LYNXEYE detector. Diffraction patterns were recorded in the 10–70° 2θ range, with a step of 0.02° and collection time of 0.3 s/step. Rietveld analysis of the patterns was carried out with a help of FullProf Suite^42^. Structure models were obtained from Crystallography Open Database^43^. The microstructure of the samples was investigated using a Carl Zeiss NVision 40 high resolution scanning electron microscope at 1 kV acceleration voltage. The IR spectra of samples were recorded on an ALPHA IR Fourier spectrometer (Brucker) in the disturbed total internal reflection mode in the range of 400–4000 cm^−1^ with a resolution of 1.5 cm^−1^.

### DNase treatment and assay

To qualitatively test functioning the DNase (I) (Pushchino Laboratories, Russia) in **B4_CaUr** medium containing 2.5 g/L of urea and 2.5 g/L CaCl_2_, the enzyme (0.25 mg/mL in B4 medium) was used to treat salmon sperm DNA (Sigma-Aldrich Co, USA) at 37 ˚C for 40 min followed by product detection by PAGE in 1% agarose gel using DNA double-stranded fragments (1 kb DNA Ladder, New England BioLabs) as markers. Products of DNase (I) treatment of salmon sperm DNA diluted in commercial DNase (I) buffer (Sigma-Aldrich CHEMIE GmbH, Germany) under the same conditions were used as a control.

To evaluate the role of eDNA in ECM formation and CaCO_3_ precipitation during the growth of *B. cereus* 4B cells in **B4_CaUr** medium, DNase (I) (0.25 mg/mL) was adding to the growth medium every 3 h during the exponential growth phase (15–69 h). Samples of the culture liquid were taken before and one hour after each enzyme addition, as well as at the end of the experiment at 44 h after the inoculation. To visualize ECM components and calcium carbonate precipitates, fluorescent microscopy was used as described above. The resulting precipitate was separated by centrifugation (4000 rpm, 5 min), washed with MilliQ water, dried, and analyzed using FTIR as described in section “CaCO_3_ particle analysis”.

### Cell-free experiment

To study the effect of DNA on calcium carbonate precipitation in cell-free system, the supernatant produced after *B. cereus* growth in the **B4** medium with the urea excess (25 g/L) was used. After the growth medium pH reached the value of 8, cells and associated ECM were separated from the supernatant (12,000 rpm, 15 min) followed by the addition of calcium chloride (25 g/L) and salmon sperm DNA (30 µg/mL) and incubation for 2 h at 37 °С. The precipitation was visually detected spectrophotometrically at 540 nm^32^. Smears from the precipitates were stained with Sytox green and studied by confocal microscope as described previously.

### Reporting summary

Further information on research design is available in the Nature Research Reporting Summary linked to this article.

## Supplementary information

Supplementary Material

Reporting Summary

## Data Availability

All data measured and analyzed during this study are included in the paper and its supplementary information file. Additional data are available from the corresponding author upon reasonable request.
