# Supplementary material for: Matrix is everywhere: extracellular DNA is a link between biofilm and mineralization in Bacillus cereus planktonic lifestyle
Source: NPJ Biofilms Microbiomes. 2023 Feb 28;9:9. doi: 10.1038/s41522-023-00377-5 (PMC9975174; doi:10.1038/s41522-023-00377-5)
Supplement: Supplementary file 1 — Supplementary Material [file 41522_2023_377_MOESM1_ESM.pdf]

Supplementary Material

Supplementary Figures

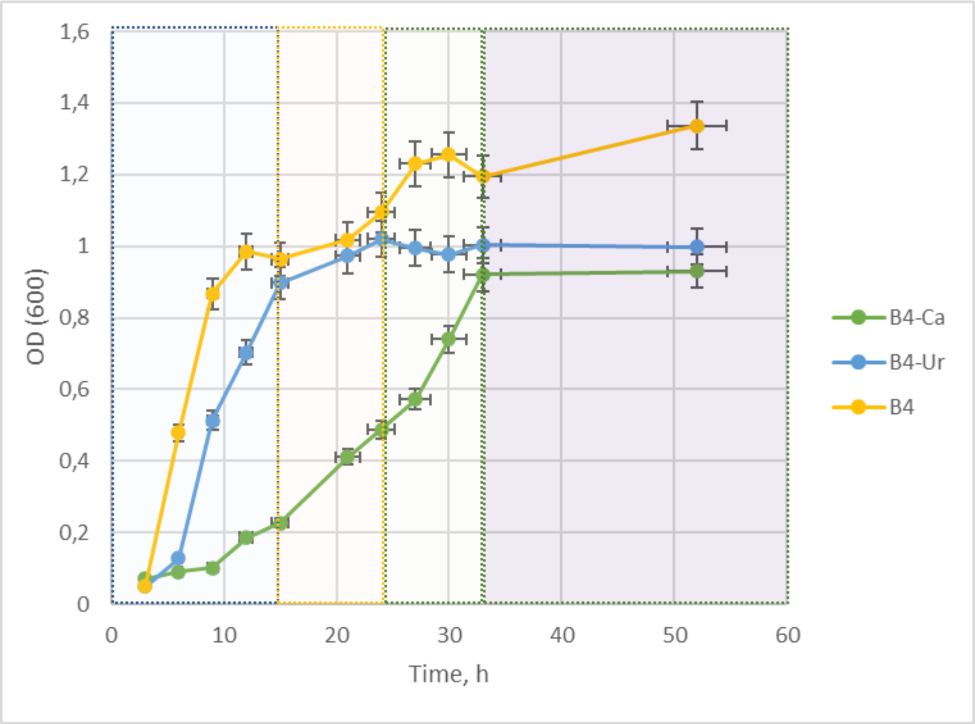

Supplementary Figure 1. Time-dependence of the bacterial biomass growth of *B. cereus* 4B in control media: **B4_Ca** (green), **B4** (yellow) **B4_Ur** (blue). Time stages are highlighted in color: 0 – 15 hours in blue; 15 – 24 hours in orange; 24 – 33 hours in green; more than 33 hours in lilac. All growth experiments were repeated 5 times (s.e.m).

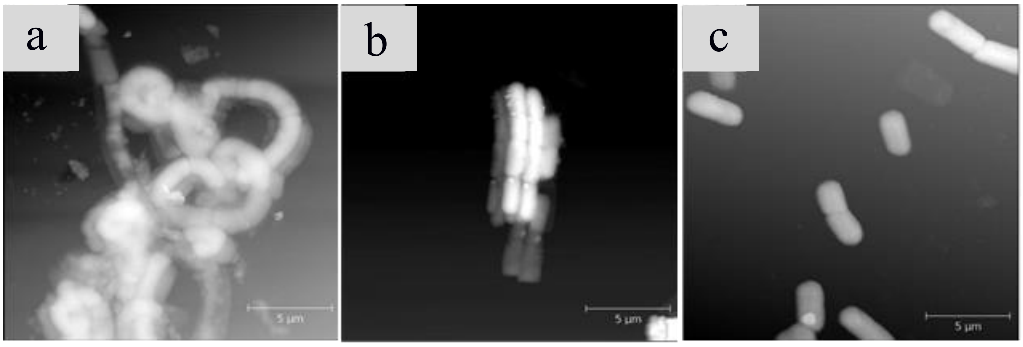

Supplementary Figure 2. AFM microscopy image of *B. cereus* after 6 hours of cultivation in media: a: B4_Ca; **b**: B4_Ur; **c:** B4. All scale bars correspond to 5 µm.

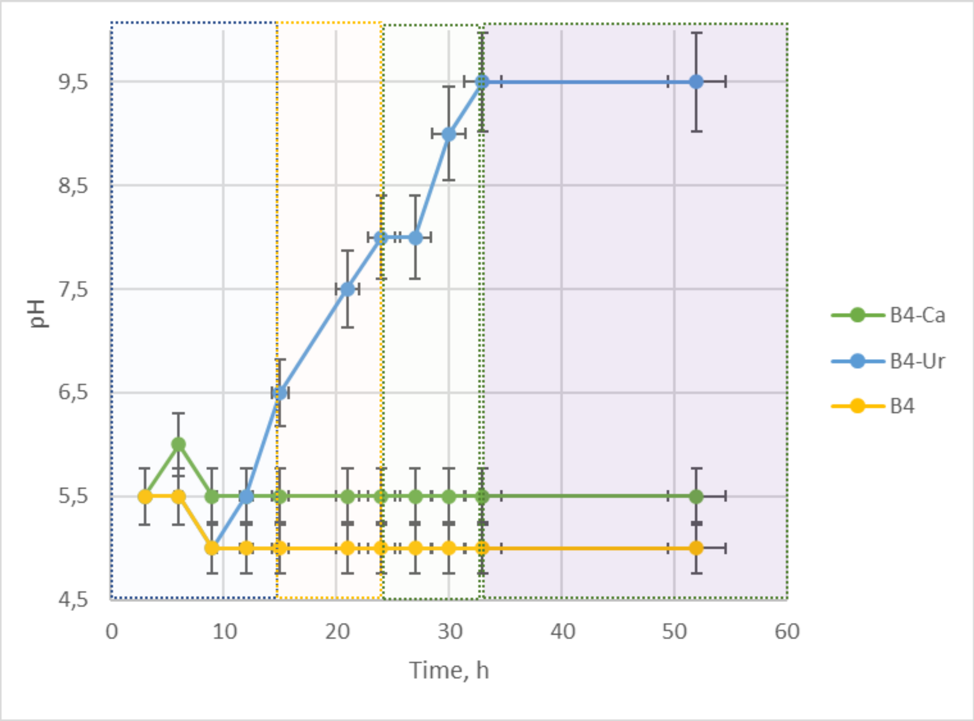

Supplementary Figure 3. Time-dependence of the medium acidity during the incubation of *B. cereus* 4B in the control media: **B4_Ca** (in green), **B4** (in yellow), **B4_Ur** (in blue). Time stages are highlighted in color: 0 – 15 hours in blue; 15 – 24 hours in orange; 24 – 33 hours in green; more than 33 hours in lilac. All growth experiments were repeated 5 times (s.e.m).

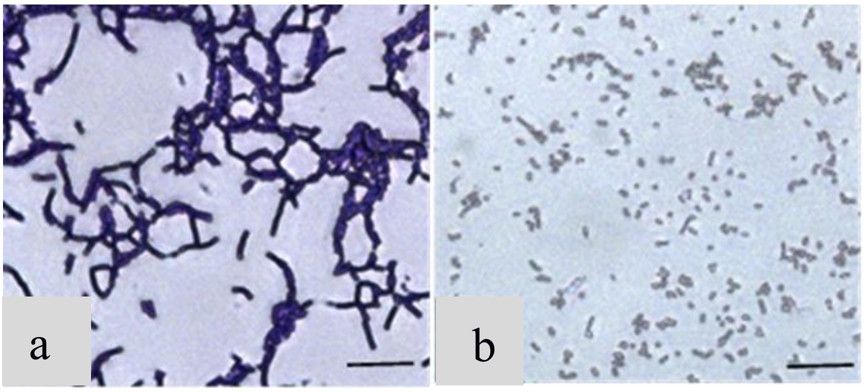

Supplementary Figure 4. Image of light microscopy with crystal violet staining of *B. cereus* cells at the fourth stage of growth (52 hours of inoculation). **a:** In the **B4_Ur** medium **b:** in the **B4** medium. All scale bars correspond to 5 µm.

.

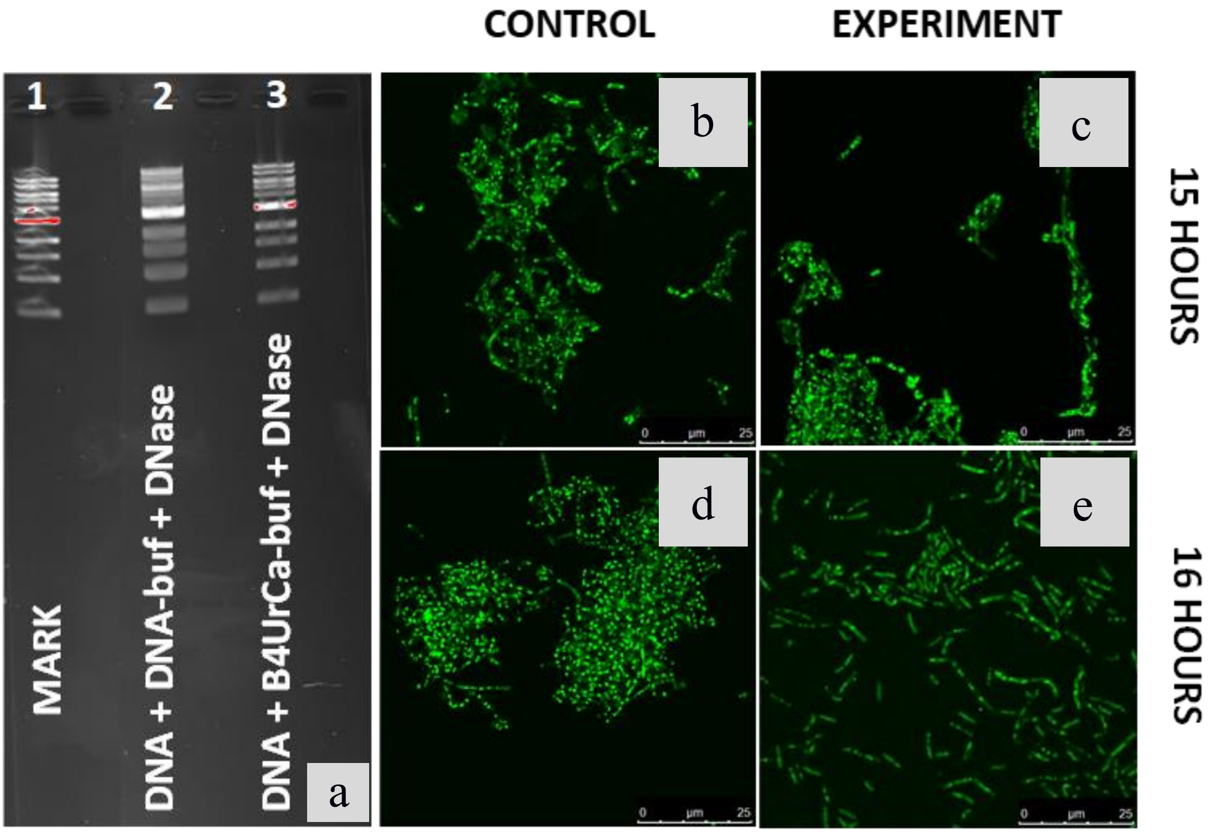

Supplementary Figure 5. DNA-DNase experiments. a: Image of agarose gel after PAGE, where **1:** DNA marker; **2:** DNA fragments obtained by hydrolysis of a salmon sperm DNA molecule with DNase (I) in a commercial DNA buffer; **3:** DNA fragments obtained by hydrolysis of a salmon sperm DNA molecule with DNase (I) in **B4-CaUr** medium. **b- e:** Confocal microscopy image with Sytox green (eDNA) staining of *B. cereus* samples in **B4_CaUr** medium before addition of the DNase (I) and cultured for 15 hours (**b, c**) and one hour after addition of DNase (I) in experimental sample (**e**) compared to a control sample without enzyme (**d**). All scale bars correspond to 25 µm.

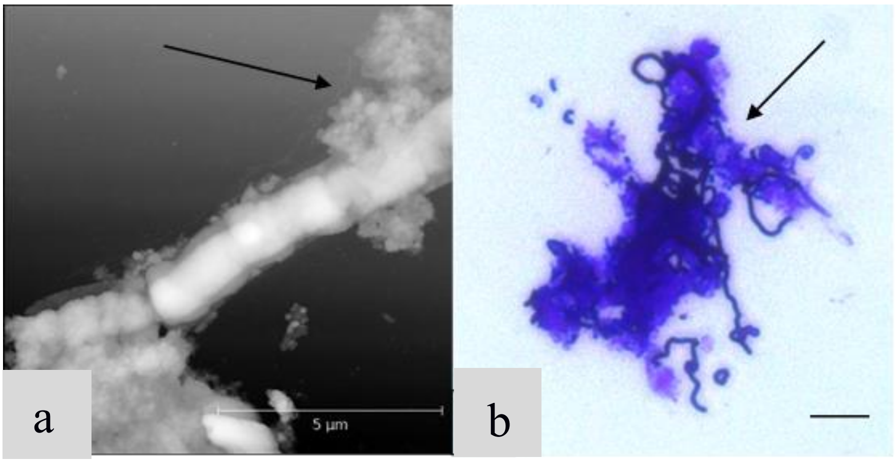

Supplementary Figure 6. a: AFM microscopy image of *B. cereus* after 9 hours of cultivation in medium **B4_Ca**. Dimensional designations correspond to 5 µm. Black arrows indicate polysaccharides. **b**: Image of light microscopy with crystal violet staining of *B. cereus* in **B4_Ca** medium at the first stage of growth (15 hours after inoculation). All scale bars correspond to 5 µm.

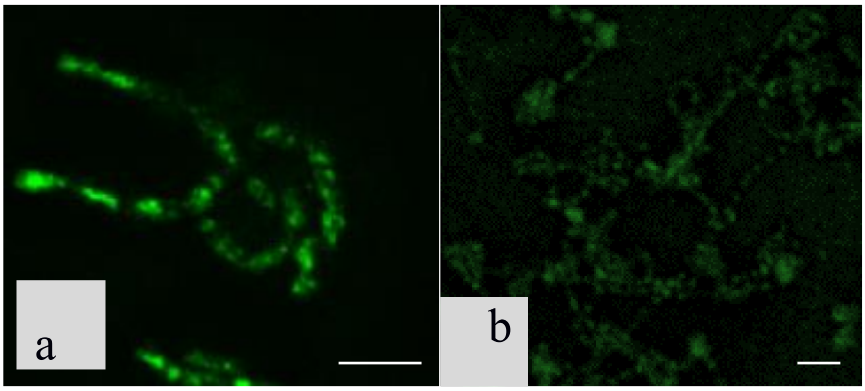

Supplementary Figure 7. Confocal microscopy image with Sytox green (eDNA) staining of *B. cereus* after 30 hours of cultivation in **B4_Ur** (**a**) and **B4_Ca** (**b**) media. All scale bars correspond to 5 µm.

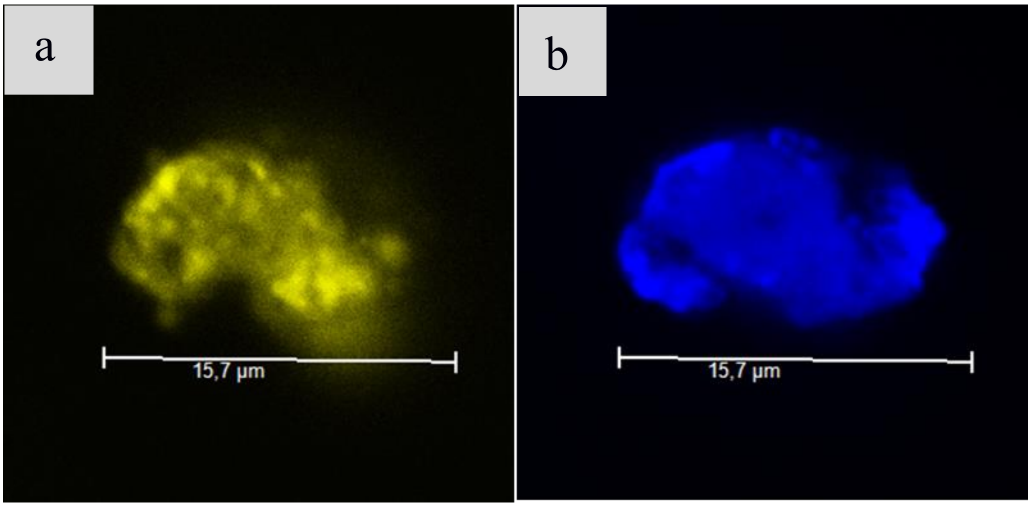

Supplementary Figure 8. Confocal microscopy images with Bromophenol blue (amyloids) staining of *B. cereus* after 68 hours of cultivation in **B4_CaUr** with DNase **a:** Confocal microscopy image with $CaCO_3$ autofluorescence. **b:** Confocal microscopy image with Bromophenol blue staining (amyloid structures).

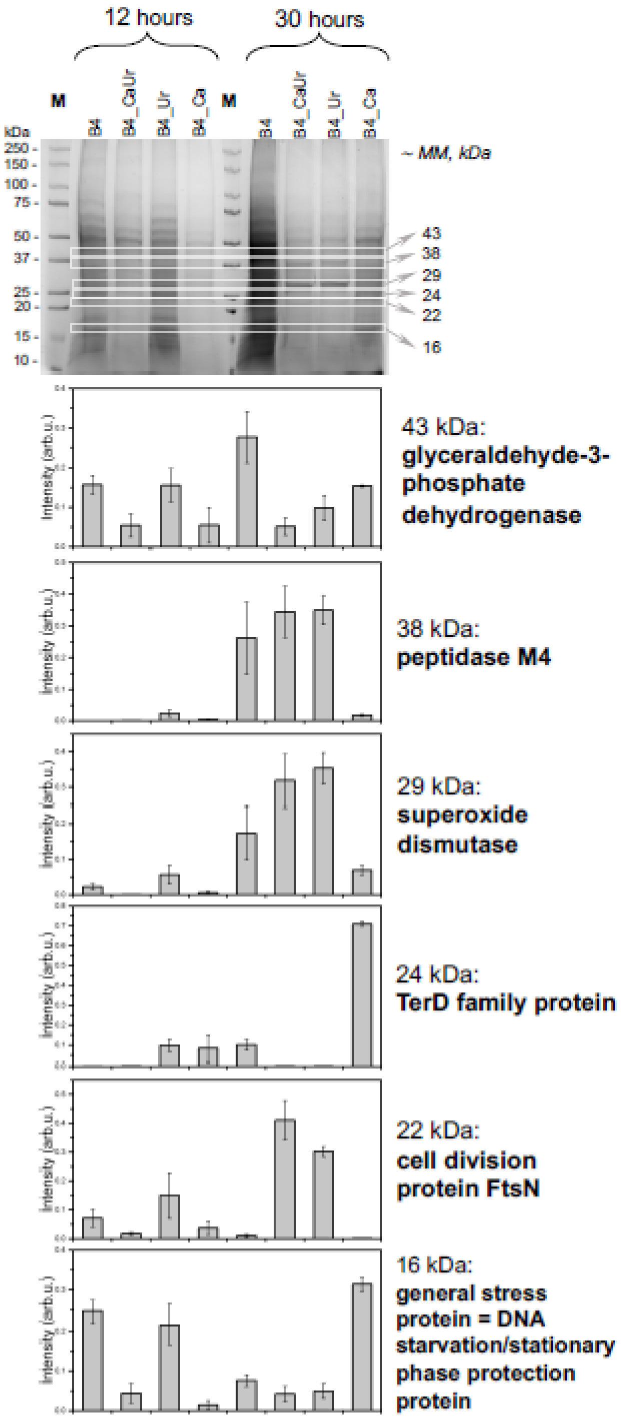

Supplementary Figure 9. Proteomic analysis of the ECM associated with cells after the growth for 12 and 30 hours in four media. At the top: an image of a protein PAGE separation of the fraction with PBS-washed cells and ECM) produced by the cells in the control **B4** and experimental media **B4_CaUr** media after 12 and 30 hours of the growth; **below:** densitometric analysis of the zones differed in color intensity (marked with arrows on the top panel). The relative volume values were obtained by normalizing the color intensity of a zone to the total color intensity of the corresponding lane.

Supplementary Methods

Proteome analysis of ECM associated with *B. cereus* cells

To isolate cell fractions associated with ECM, 2-mL aliquots were withdrawn from the 100-mL flasks containing **B4, B4_Ca, B4_Ur,** or **B4_CaUr** media at 12 and 30 hours of the growth at 37°C with shaking at 110 rpm. The number of cells was estimated by optical absorption at 600 nm using spectrophotometer Hitachi U-3310. Each sample was centrifuged at 8000 *g* for 10 minutes. Fractions containing the pellets were re-dissolved in 2 mL of PBS, centrifuged for 10 minutes at 8000 *g,* and freezed at -20°C. After thawing cell content was used for electrophoretic separation of proteins for further proteome analysis.

Samples of PBS-washed bacteria pellet rich of ECM were mixed with Laemmli's buffer containing β-mercaptoethanol [1], denatured at 99°C for 5 minutes, and loaded into the wells of a polyacrylic gradient gel (8-16%) using a Kaleidoscope kit (BioRad) as a molecular weight marker. Fragments of the Coomassie-stained gel were excised and prepared for mass-spectrometric analysis as described in [2]. Briefly, the gel fragments were washed from the dye twice with 30 mM NH_4HCO_3, 40% acetonitrile, after that it was dehydrated with 100% acetonitrile, and treated with trypsin (Promega) (20 µg/mL in 50 mM NH_4HCO_3) at 37°C for 5 hours. Tryptic peptides were mixed with a 2,5-DHB matrix (Bruker), applied to a target, and mass spectra were obtained on a MALDI-TOF/TOF mass spectrometer UltrafleXtreme (Bruker) in the positive ion mode. For each spectrum, 5000 laser pulses were summed up. Protein identification was performed using MASCOT (www.matrixscience.com) and the NCBI database (www.ncbi.nlm.nih.gov). The error was limited to 20 ppm. Methionine oxidation and deamidation were indicated as variable modifications. Identification was considered reliable ($p < 0.05$) if the score value exceeded the threshold value.

Supplementary notes

1. To analyze differences in the proteome composition in ECM associated with *B. cereus* cells grown in the control (**B4_Ur**, **B4_Ca** and **B4**) and the **B4_CaUr** media over time, the protein fractions obtained from PBS-washed cells containing extracellular matrix were analyzed by PAGE separation (**Fig. 9**) followed by mass-spectrometric identification and densitometric analysis. Analysis of the differences in the position and intensity of the zones after one-dimensional electrophoresis at 12 and 30 hours (growth periods 1 and 3) for bacteria grown in different media made it possible to identify a number of major differing proteins. Mass spectrometry in samples from the **B4_CaUr** medium and the **B4_Ur** medium at the 3rd period identified various isoforms of the FtsN protein and magnesium superoxide dismutase, as well as extracellular metalloprotease M4. In samples of bacteria grown in the **B4_Ca** medium proteins of the TerD family (calcium-binding membrane proteins) and DNA starvation/stationary phase protection proteins were detected.

2. To analyze the potential of dead *B. cereus* 4B cells in MICP, cells were grown in the basic **B4_CaUr** medium for 10 hours, then treated with the gamma radiation at a dose of 20 Gray followed by incubation at 37°C for 52 hours with visual monitoring.

Supplementary references

[1] U. K. LAEMMLI, “Cleavage of Structural Proteins during the Assembly of the Head of Bacteriophage T4,” *Nature*, vol. 227, no. 5259, pp. 680–685, Aug. 1970, doi: 10.1038/227680a0.

[2] O. I. Antimonova *et al.*, “Changing times: Fluorescence-lifetime analysis of amyloidogenic SF-IAPP fusion protein,” *J Struct Biol*, vol. 205, no. 1, pp. 78–83, Jan. 2019, doi: 10.1016/j.jsb.2018.11.006.
