# Supplementary material for: Matrix is everywhere: extracellular DNA is a link between biofilm and mineralization in Bacillus cereus planktonic lifestyle
Source: NPJ Biofilms Microbiomes. 2023 Feb 28;9:9. doi: 10.1038/s41522-023-00377-5 (PMC9975174; doi:10.1038/s41522-023-00377-5)
Supplement: Supplementary file 2 — Reporting Summary [file 41522_2023_377_MOESM2_ESM.pdf]

nature portfolio

Corresponding author(s): Anna A. Kulminskaya

Last updated by author(s): 17/01/23

Reporting Summary

Nature Portfolio wishes to improve the reproducibility of the work that we publish. This form provides structure for consistency and transparency in reporting. For further information on Nature Portfolio policies, see our Editorial Policies and the Editorial Policy Checklist.

Statistics

For all statistical analyses, confirm that the following items are present in the figure legend, table legend, main text, or Methods section.

n/a	Confirmed	
☐	☒	The exact sample size (*n*) for each experimental group/condition, given as a discrete number and unit of measurement
☐	☒	A statement on whether measurements were taken from distinct samples or whether the same sample was measured repeatedly
☐	☒	The statistical test(s) used AND whether they are one- or two-sided<br>*Only common tests should be described solely by name; describe more complex techniques in the Methods section.*
☐	☒	A description of all covariates tested
☐	☒	A description of any assumptions or corrections, such as tests of normality and adjustment for multiple comparisons
☒	☐	A full description of the statistical parameters including central tendency (e.g. means) or other basic estimates (e.g. regression coefficient) AND variation (e.g. standard deviation) or associated estimates of uncertainty (e.g. confidence intervals)
☒	☐	For null hypothesis testing, the test statistic (e.g. *F*, *t*, *r*) with confidence intervals, effect sizes, degrees of freedom and *P* value noted<br>*Give P values as exact values whenever suitable.*
☒	☐	For Bayesian analysis, information on the choice of priors and Markov chain Monte Carlo settings
☒	☐	For hierarchical and complex designs, identification of the appropriate level for tests and full reporting of outcomes
☒	☐	Estimates of effect sizes (e.g. Cohen's *d*, Pearson's *r*), indicating how they were calculated

Our web collection on statistics for biologists contains articles on many of the points above.

Software and code

Policy information about availability of computer code

Data collection	-
Data analysis	-

For manuscripts utilizing custom algorithms or software that are central to the research but not yet described in published literature, software must be made available to editors and reviewers. We strongly encourage code deposition in a community repository (e.g. GitHub). See the Nature Portfolio guidelines for submitting code & software for further information.

Data

Policy information about availability of data

All manuscripts must include a data availability statement. This statement should provide the following information, where applicable:

- Accession codes, unique identifiers, or web links for publicly available datasets
- A description of any restrictions on data availability
- For clinical datasets or third party data, please ensure that the statement adheres to our policy

All data measured and analyzed during this study are included in the paper and its supplementary information file

Human research participants

Policy information about studies involving human research participants and Sex and Gender in Research.

Reporting on sex and gender	-
Population characteristics	-
Recruitment	-
Ethics oversight	-

Note that full information on the approval of the study protocol must also be provided in the manuscript.

Field-specific reporting

Please select the one below that is the best fit for your research. If you are not sure, read the appropriate sections before making your selection.

☐ Life sciences ☐ Behavioural & social sciences ☒ Ecological, evolutionary & environmental sciences

For a reference copy of the document with all sections, see nature.com/documents/nr-reporting-summary-flat.pdf

Ecological, evolutionary & environmental sciences study design

All studies must disclose on these points even when the disclosure is negative.

Study description	In the proposed article, we describe a study of the mutual influence of two processes: biomineralization and biofilm formation on a model planktonic bacterium B. cereus. The study is carried out on experimental media containing excess calcium and urea (without and with the addition of DNase), as well as on three control media. The growth of biomass, changes in the pH of the medium, the presence of macromolecules of the extracellular matrix (eDNA and amyloids), as well as the appearance of a precipitate of calcium carbonate in the process of biomineralization were monitored. The role of eDNA in the process of biomineralization is partially shown. All experiments were repeated 5 times.
Research sample	Aliquots of B. cereus cultural liquid cultivated in various control and experimental media became the research samples in the experiment.
Sampling strategy	The experiment was completely carried out independently anew 5 times
Data collection	The strain Bacillus cereus 4B (an undomesticated prototrophic strain from our laboratory collection) deposited at the National Bioresource Center “All-Russian Collection of Industrial Microorganisms” under the number B-14265
Timing and spatial scale	Each of the experiments was carried out from 52 to 68 hours, during which aliquots of the culture liquid were taken for research every 3 hours.
Data exclusions	We have no excluded data. Data not included in the main text of the article has been added to the Supplementary Materials.
Reproducibility	Attempts to repeat the experiment were successful and included in the article.
Randomization	Different groups of samples in the experiment were due to the different composition of the culture medium in which B. cereus was grown, the groups were not random.
Blinding	There were no blinding reaserch in the experiment.

Did the study involve field work? ☐ Yes ☒ No

Reporting for specific materials, systems and methods

We require information from authors about some types of materials, experimental systems and methods used in many studies. Here, indicate whether each material, system or method listed is relevant to your study. If you are not sure if a list item applies to your research, read the appropriate section before selecting a response.

Materials & experimental systems

n/a	Involved in the study
☒	☐ Antibodies
☒	☐ Eukaryotic cell lines
☒	☐ Palaeontology and archaeology
☒	☐ Animals and other organisms
☒	☐ Clinical data
☒	☐ Dual use research of concern

Methods

n/a	Involved in the study
☒	☐ ChIP-seq
☒	☐ Flow cytometry
☒	☐ MRI-based neuroimaging
